# Study on Regulation Mechanism of Heat Transport at Aluminum Nitride/Graphene/Silicon Carbide Heterogeneous Interface

**DOI:** 10.3390/nano15120928

**Published:** 2025-06-14

**Authors:** Dongjing Liu, Pengbo Wang, Zhiliang Hu, Jia Fu, Wei Qin, Jianbin Yu, Yangyang Zhang, Bing Yang, Yunqing Tang

**Affiliations:** 1 Guangxi Key Laboratory of Manufacturing System & Advanced Manufacturing Technology, School of Mechanical and Electrical Engineering, Guilin University of Electronic Technology, Guilin 541004, China; ldj168168@guet.edu.cn (D.L.); wpb139139@mails.guet.edu.cn (P.W.); huzhiliang@mails.guet.edu.cn (Z.H.); 2 School of Mechanical Engineering, Shandong University, Jinan 250061, China; fujia@mail.sdu.edu.cn; 3 State Key Laboratory of Advanced Equipment and Technology for Metal Forming, Shandong University, Jinan 250061, China; 4 Key Laboratory of High Efficiency and Clean Mechanical Manufacture of Ministry of Education, Jinan 250061, China; 5 SDEE Contemporary Energy Technology Co., Ltd., Jinan 250061, China; qinwei@scetl-cee.com.cn (W.Q.); yujianbin@scetl-cee.com.cn (J.Y.); zhangyangyang@scetl-cee.com.cn (Y.Z.); 6 Centre for Advanced Laser Manufacturing (CALM), School of Mechanical Engineering, Shandong University of Technology, Zibo 255000, China

**Keywords:** interfacial thermal conductivity, phonon density of states, temperature effects, size effects, vacancy defects

## Abstract

In order to solve the self-heating problem of power electronic devices, this paper adopts a nonequilibrium molecular dynamics approach to study the thermal transport regulation mechanism of the aluminum nitride/graphene/silicon carbide heterogeneous interface. The effects of temperature, size, and vacancy defects on interfacial thermal conductivity are analyzed by phonon state density versus phonon participation rate to reveal their phonon transfer mechanisms during thermal transport. It is shown that the interfacial thermal conductance (ITC) increases about three times when the temperature increases from 300 K to 1100 K. It is analyzed that the increase in temperature will enhance lattice vibration, enhance phonon coupling degree, and thus increase its ITC. With the increase in the number of AlN-SiC layers from 8 to 28, the ITC increases by about 295.3%, and it is analyzed that the increase in the number of AlN-SiC layers effectively reduces the interfacial scattering and improves the phonon interfacial transmission efficiency. The increase in the number of graphene layers from 1 layer to 4 layers decreases the ITC by 70.3%. The interfacial thermal conductivity reaches a minimum, which is attributed to the increase in graphene layers aggravating the degree of phonon localization. Under the influence of the increase in graphene single and double vacancy defects concentration, the ITC is slightly reduced. When the defect rate reaches about 20%, the interfacial thermal conductance of SV (single vacancy) and DV (double vacancy) defects rises back to 5.606 × 10^−2^ GW/m^2^K and 5.224 × 10^−2^ GW/m^2^K, respectively. It is analyzed that the phonon overlapping and the participation rate act at the same time, so the heat-transferring phonons increase, increasing the thermal conductance of their interfaces. The findings provide theoretical support for optimizing the thermal management performance of heterostructure interfaces.

## 1. Introduction

With the advancement of nano-micromachining technology, power electronic devices are developing in the direction of miniaturization, high power, and high integration, resulting in a sharp increase in internal heat flow density, which generates problems such as poor reliability and short life span, affecting the stable operation of power electronic devices [1,2,3,4,5,6]. Heterogeneous interface materials [7,8,9] have been widely recognized as the dielectric layer connecting the chip to the heat sink, with third-generation semiconductors, led by GaN, emerging as an essential material for addressing the self-heating problem of power electronics, and SiC standing out among several commonly used substrates due to its excellent chemical and thermal conductivity properties. However, a significant lattice mismatch exists between the GaN epitaxial layer and the SiC substrate [10]. An AlN film is frequently epitaxialized as a buffer layer before growing the GaN film during production, making AlN/SiC a significant component of GaN-based power electronics [11].

In addition, graphene has been widely introduced into heterostructures as an intermediate dielectric layer by its ultra-high in-plane thermal conductivity and flexible interfacial coupling properties, and its core role is reflected in the lattice mismatch buffering and phonon transport modulation, which in turn enhances the interfacial thermal conductivity. For example, Hu et al. [12] added graphene to GaN/SiC heterostructures to significantly increase the interfacial thermal conductance and heavily activate its phonons for interfacial heat transport; Yang et al. [13] did the same to GaN/SiC heterostructures, increasing the interfacial thermal conductance by 46.5%; and Tao et al. [14] added a graphene buffer layer to GaN/diamond heterostructures to make the interfacial thermal conductance rise by 33%. It is shown that graphene further reduces the lattice mismatch and thermal mismatch between AlN and SiC, which is conducive to the heat dissipation at the interface of AlN/SiC heterostructures and significantly suppresses the “self-heating effect” inside the structure.

In recent years, many efforts have been made on the interfacial thermal properties of AlN/graphene/SiC heterostructures. Yang’s team [11,15] reduced the interfacial thermal resistance (ITR) of w-AlN/3C-SiC heterojunctions by two strategies. First, a graphene interlayer with 8% doping concentration was inserted into the w-AlN/3C-SiC heterostructure, which utilized Si doping to enhance the strong interaction between 3C-SiC and silicon-doped graphene (SiG), resulting in a reduction of the ITR by 31.72%. Second, polycrystalline graphene was introduced into the w-AlN/3C-SiC, which resulted in an increase of the ITC of about 75.09%. Yang’s [16] research team also investigated the effect of nanoscale nonplanar feature structures (NNFS) on the interfacial thermal resistance (ITR) of w-AlN/bilayer graphene/3C-SiC heterojunction by molecular dynamics system. It is found that the randomly distributed NNFSS can effectively reduce the ITR with a maximum reduction of 18.1% in the frequency domain range of 0–18 THz. Tian et al. [17] investigated the ITC of AlN/SiC interfaces by nonequilibrium molecular dynamics simulations, and the introduction of an amorphous layer at the AlN/SiC interface will lead to an increase in its ITC by a factor of 2.32. In addition, advanced precision preparation techniques for heterostructures provide strong support for revealing the interfacial thermal conduction mechanism. Zakaria et al. [18] demonstrated the nucleation process of AlN grown on graphene substrate by gas-phase deposition; Xu et al. [19] also grew aluminum nitride thin films on multilayer graphene/silicon carbide by hydride gas-phase epitaxy at a high temperature of 1100 °C.

Researchers are currently concentrating on studying the heterostructures of two materials made of graphene with silicon carbide, gallium nitride, and aluminum nitride [9,17,20,21]. Examples of these are graphene/GaN [20], graphene/SiC [9], GaN/AlN [21], and AlN/SiC [17], among others. Nevertheless, nothing is known about the triple-layered heterostructure of the heat transport regulating mechanism of silicon carbide, graphene, and aluminum nitride. As a result, the purpose of this paper is to systematically investigate the interfacial thermal conductivity properties of aluminum nitride/graphene/silicon carbide heterostructures using analytical tools such as phonon density of states, overlapping factor S, and phonon participation rate, while taking into account several influencing factors such as temperature, size, and heterostructure interfacial defects. The results of this work provide a theoretical foundation and experimental support for the creation of high-performance thermal interface materials and thermal management technology for power electronic devices.

## 2. Materials and Methods

### 2.1. Modeling

**Figure 1:**
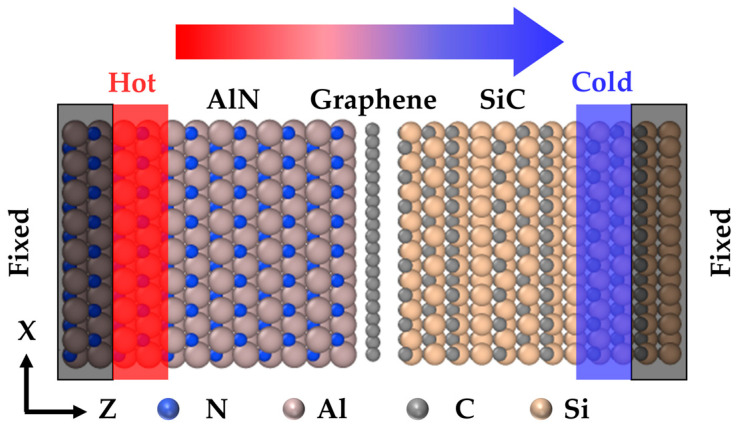
Interface model of aluminum nitride/graphene/silicon carbide heterostructure.

4H-SiC has a more wide application in devices and has a better interfacial thermal contact with graphene compared to 3C-SiC and 6H-SiC [9], so here we adopt 4H-SiC to construct the sandwich heterostructure with graphene and w-AlN. The software Material Studio 2023 is used to build the AlN/graphene/SiC heterostructure model, and the model is idealized as a structure without impurities or defects, as shown in Figure 1. Table 1 shows the lattice parameters required for modeling.

To reduce the effect of interfacial lattice mismatch, the model size of AlN after cell expansion in the x-, y-, and z-directions of the pristine cell is 37.932 Å × 25.029 Å × 30.101 Å. Graphene is a monolayer planar model that solely considers the x- and y-directions. The increased cell size is 38.346 Å × 24.6 Å. The obtained cell size following SiC expansion is 37.319 Å × 24.624 Å × 30.138 Å. After cell growth, due to the differing coefficients of thermal expansion of the two materials in the heterostructure, the lattice mismatch rate was introduced [22] with the following equation:

(1) $$f=\left|\frac{\alpha_{1}-\alpha_{2}}{\alpha_{1}}\right|,$$

where $\alpha_{1}$ is the substrate lattice constant and $\alpha_{2}$ is the epitaxial film lattice constant. Aluminum nitride/graphene and silicon carbide/graphene had lattice mismatch rates of 1.1% and 2.7% in the x-direction, respectively, and 1.7% and 0.1% in the y-direction, with lattice mismatches of less than 5% in both directions.

### 2.2. Simulation Process

The simulation process of the study was all carried out using Lammps (2 Aug 2023 stable release) [23], the open visualization tool (OVITO 3.12.4) [24] was used to visualize the simulations, and the interactions between the atoms of the aluminum nitride material during the simulations were described by the Tersoff potential function [25]. The interactions between the atoms of the silicon carbide were described by the Tersoff–Erhart–Albe (TEA) potential function [26], and the interaction between graphene atoms was described by the AIREBO potential function [27]. These potentials [25,26,27] have been widely used and validated for similar systems in many studies. Finally, the interaction of atoms between different materials is described by using the Lennard-Jones (L-J) potential, which is expressed by the L-J potential function

(2) $$V\left(r_{ij}\right)=4\chi \varepsilon_{ij}\left[{\left(\sigma /r_{ij}\right)}^{12}-{\left(\sigma /r_{ij}\right)}^{6}\right],$$

where $r_{ij}$ represents the distance between atom *i* and atom *j*; χ represents the scaling factor of the interfacial coupling strength; $\varepsilon_{ij}$ represents the interaction strength between atom *i* and atom *j*; and represents the atomic spacing with zero potential energy. The relevant L-J potential function parameters [11,28] are shown in Table 2 below.

All three directions of the model are set as periodic boundary conditions [29]. Under the shrinkable boundary condition in the z-direction, the simulation results differ from those under periodic boundary conditions by less than 5%. Firstly, two layers of atoms are fixed at each end of the model, and secondly, the two layers of atoms adjacent to the fixed layers are set as cold and hot regions, respectively, with the positive direction of the z-axis as the direction of heat transport in the heterostructure, as shown in Figure 1.

**Figure 2:**
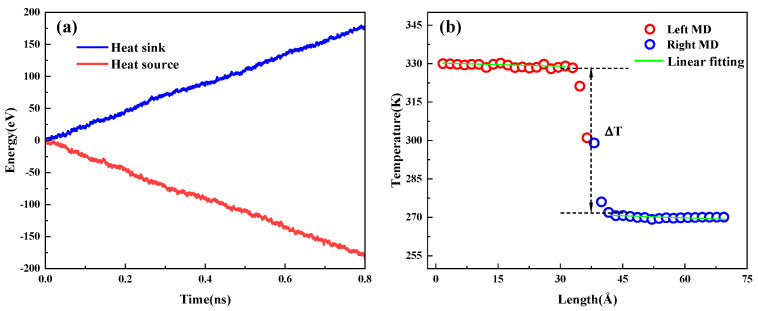
(**a**) Energy profile; (**b**) temperature gradient.

According to the previous research of our group [30], the time step is set to 0.25 fs during simulation, and the velocity Verlet integration method is used to integrate the equations of motion. The simulation process firstly optimizes the heterogeneous structure, and the structural energy is minimized by the conjugate gradient algorithm, and then the NVT (Canonical Ensemble) systematic relaxation is carried out at 300 K for 0.25 ns and is transferred to NVE (Microcanonical Ensemble) systematic, eliminating the heat bath perturbation. In the nonequilibrium simulation stage, a Langevin [31] heat bath at 330 K and 270 K was applied at the axial ends of the system to establish a steady-state temperature gradient. Real-time acquisition of temperature field distribution through spatial block division technology (divided into 40 statistical units along the Z-direction), followed by averaging the acquired temperature data samples every 1000 steps and combining with 0.75 ns the average temperature of data to compute the heat flow density and thus the temperature gradient, as shown in Figure 2b. According to the energy dynamic distribution of the heat source-sink system in Figure 2a, the heat flux values were obtained based on the calculation of the slope of the fitted curve, while the temperature abruptness at the interface was determined by the linear regression analysis of the temperature distribution on both sides. Finally, the interfacial thermal conductance is calculated based on Fourier’s law [32]:

(3) K=q/ΔT,

where *q* is the heat flow density and Δ*T* is the temperature difference at the interface. The heat flow density *q* is calculated from the energy exchanged between the system and the heat bath

(4) $$q=\frac{\left(\partial E_{hot}/\partial t+\partial E_{cold}/\partial t\right)}{2A},$$

where *∂E*/*∂t* is the rate of energy change and A is the cross-sectional area perpendicular to the direction of heat flow.

The velocity autocorrelation function (VACF) of an atom can be calculated by the following equation:

(5) $$VACF\left(t\right)=\frac{1}{N}\sum_{i=1}^{N}\langle v_{i}\left(0\right)v_{i}\left(t\right)\rangle ,$$

where $V_{i}(t)$ represents the velocity vector of the ith particle at time t, *N* is the number of atoms in the region, and < > denotes statistical averaging. The density of phonon states (PDOS) can be calculated by the Fourier transform of the *VACF* [33]

(6) $$P\left(\omega \right)=\int_{-\infty }^{\infty }e^{i\omega t}VACF\left(t\right)dt,$$

where $P_{Gr}(\omega )$ is the PDOS at frequency ω to reveal the thermal transport mechanism at the interface of aluminum nitride/graphene/silicon carbide heterostructure.

In the interface model, all phonons impinging on the interface undergo elastic scattering and enter the neighboring medium. The probability of phonon transmission is proportional to the density of phonon states in each medium. By calculating and analyzing the region of overlap of the phonon density of states (PDOS), the transport properties of phonons passing through an interface by elastic scattering can be evaluated. Specifically, the overlapping factor *S* [34] is used to quantify how well the phonon spectra match

(7) $$S=3\int min\left\{P_{AlN}\left(\omega \right),P_{Gr}\left(\omega \right),P_{SiC}\left(\omega \right)\right\}d\omega ,$$

where $P_{AlN}(\omega )$, $P_{Gr}(\omega )$, $P_{SiC}(\omega )$ correspond to the PDOS of aluminum nitride, graphene, and silicon carbide, respectively, the more enormous value of the overlapping factor *S* indicates that the phonon vibration modes at the interface are more coordinated, the degree of phonon coupling is higher, and the interfacial thermal conductance is elevated accordingly.

Phonon participation ratio (*PPR*) [35] is often used to analyze interfacial heat transport and is an effective method to analyze the degree of phonon localization, as calculated by the following equation:

(8) $$PPR\left(\omega \right)=\frac{1}{N}\frac{{\left[\sum_{j}P_{n}^{2}\left(\omega \right)\right]}^{2}}{\sum_{j}P_{n}^{4}\left(\omega \right)},$$

where *N* is the total number of atoms and $P_{n}(\omega )$ is the number of atoms at the corresponding frequency PDOS. *PPR* = 0.4 is considered the discriminant between the delocalized and localized modes [36]. When *PPR*(*ω*) < 0.4, the phonon is in the localized mode, and phonons in the localized domain do not participate in thermal transport; conversely, when *PPR*(*ω*) > 0.4, the phonons are in delocalized mode, and most of the phonons in the delocalization are free, are the main contributors to phonon thermal transport. By judging the state of the phonons in each frequency domain, the changing condition of the interfacial thermal conductivity can be revealed.

## 3. Results

### 3.1. Temperature Effects

**Figure 3:**
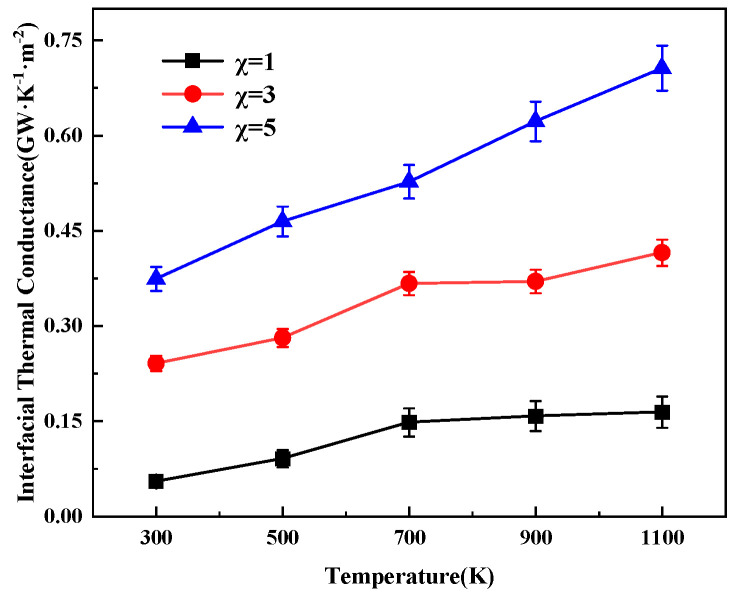
Plot of interfacial thermal conductance versus temperature for different coupling strengths.

In this section, the effect of temperature on the interfacial thermal conductance of heterostructures is investigated. The study model is set as 12 layers each of aluminum nitride and silicon carbide, with 1 layer of graphene inserted in the middle, and the interfacial thermal conductance is calculated for the temperature range of 300–1100 K. The results are shown in the following Figure 3. The results show that the interfacial thermal conductance increases with increasing temperature when the coupling strength is 1, 3, and 5, respectively. This is due to the fact that high temperature enhances the Umklapp scattering effect [37], which promotes the participation of high-frequency phonon channels and thus increases the number of thermally transported phonons. This conclusion is consistent with the findings of Wu et al. [38] on graphene/MoS_2_ heterostructures and Hong et al. [39] on phosphine/graphene bilayers, which show that increased temperature enhances the ITC. Specific data show that the interfacial thermal conductance at a coupling strength of 1 increases from 5.5096 × 10^−2^ GW/m^2^K at 300 K to 1100 K to 1.64295 × 10^−1^ GW/m^2^K. When the temperature is fixed at 300 K, increasing the coupling strength from 1 to 5 raises the interfacial thermal conductance to 5.8 times the original value. It is analyzed that the increase in coupling strength enhances the contact pressure, which strengthens the phonon coupling at the heterogeneous interface [39], leading to an increase in ITC.

**Figure 4:**
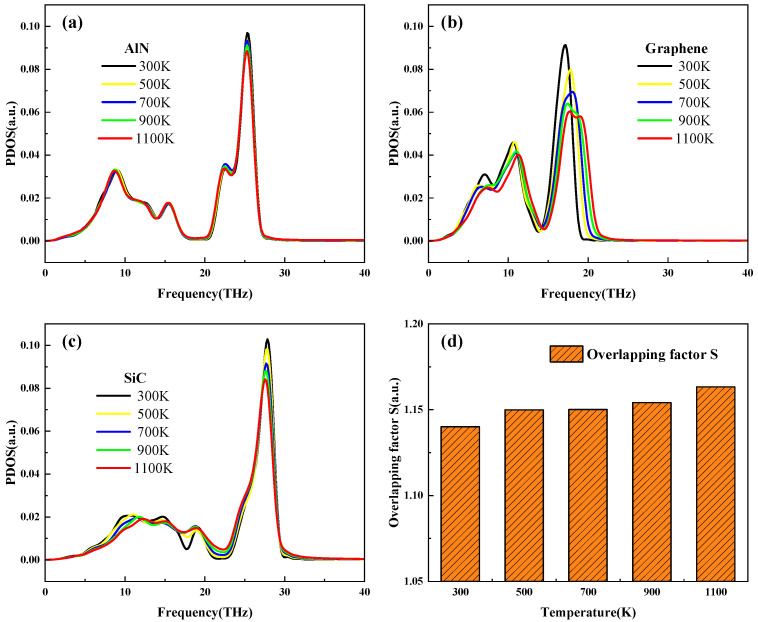
Temperature-activated PDOS profiles of (**a**) AlN, (**b**) graphene, and (**c**) SiC; (**d**) overlapping factor *S*.

To further investigate the reason for the variation of interfacial thermal conductance with temperature, this study analyzes it by means of the PDOS and the overlapping factor *S*. AlN’s PDOS curves are displayed in Figure 4a. The PDOS curves of graphene and SiC at temperature rise are shown in Figure 4b,c; from the graphene PDOS curves, the PDOS has a value only at 0–22 THz, which suggests that the phonon transport occurs in this frequency domain. Thus, only this interval of the PDOS is analyzed. Figure 4a demonstrates that the PDOS curves of AlN are highly overlapped at different temperatures, indicating that its phonons are insensitive to temperature changes. Graphene’s PDOS curves show two peaks at about 10 THz and 17 THz, with the peak at about 10 THz decreasing with an overall shift to the right as the temperature rises, forming a trough at 14 THz. The PDOS curves then show a significant change to the right as the temperature increases from 18 THz to about 21 THz, which occupies a wider frequency domain and makes the graphene with the AlN and SiC overlap region more significant. This helps to improve the interfacial thermal transport capacity. The value of the overlapping factor S is shown in Figure 4d, and it can be seen that the value of S increases with the increase in temperature. It is analyzed that the higher the value of the overlapping factor S, the matching number of phonons starts to grow, which enhances their coupling strength and thus enhances the interfacial heat transport capacity. The interfacial thermal conductance shows an overall upward trend with temperature increase.

### 3.2. Dimensional Effects

When the dimensions of the material are changed, the heat flow will change, which will trigger the size effect at the heterogeneous interface. This section focuses on the size effect’s influence on the heterostructure’s heat transport. Therefore, the model only changes the thickness (z-direction length). To improve the stability of the heterostructure, the model with the same number of AlN and SiC layers is constructed.

**Figure 5:**
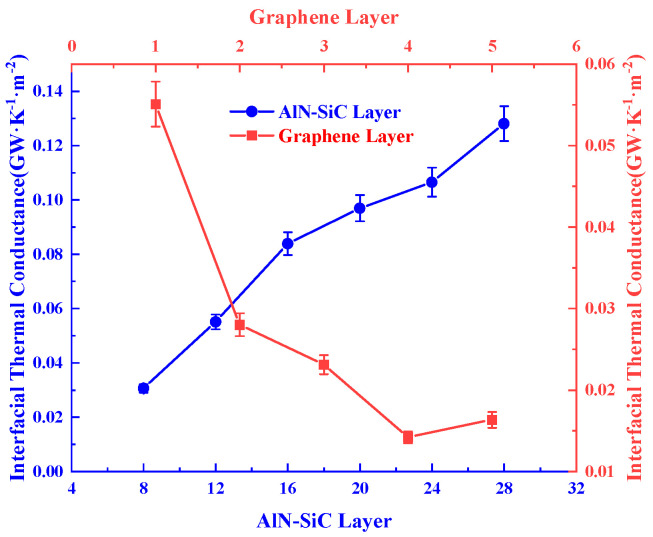
Interfacial thermal conductance under size effect.

As seen in Figure 5, when the graphene is kept constant at a single layer, the ITC increases significantly with the increase of AlN-SiC thickness, and the ITC increases from 3.0598 × 10^−2^ GW/m^2^K at 8 layers to 1.28096 × 10^−1^ GW/m^2^K at 28 layers, while when the heterostructure is fixed at a constant level of 12 layers on both sides, the ITC increases with the increase of graphene dimensions from 1 layer to 5.5096 × 10^−2^ GW/m^2^K, firstly decreases to 1.4193 × 10^−2^ GW/m^2^K at 4 layers, and then slowly increases to 1.6343 × 10^−2^ GW/m^2^K at 5 layers. The change trend is consistent with the results of many scholars [36,40]. For the variation of AlN-SiC dimensions, it is also mentioned in related studies, such as Md Shafkat et al. [41], that the interfacial thermal resistance decreases gradually with the increase of the thickness of AlN. In addition, Watari K et al. [42] calculated the mean free path (MFP) of AIN, and the room temperature MFP of AlN ceramics and crystals was calculated to be more than 40 nm. The comparison found that the size of AlN in the modelling is much smaller than the MFP, so the phonons are easily scattered with the interface in the system smaller than its MFP, and the thicker AlN film may form a more complete crystal structure, which reduces the interfacial defects and inelastic scattering, and thus facilitates the efficiency of the phonon transmission across the interface [43]. However, for the interfacial structure, since its thickness is much smaller than the feature size of the adjoining films, the effect of the film thickness-induced reduction in phonon MFP on interfacial thermal conductance remains unclear. Therefore, here, we investigated the influence of the thickness of AlN and SiC films on the ITC between them, and the results show that the ITC increases progressively with increasing AlN-SiC dimensions. For the change of graphene, it is analyzed that the thermal conductivity of graphene approaches that of bulk graphite after the number of graphene layers exceeds four [44], so the interfacial thermal conductivity decreases first and then increases slowly.

**Figure 6:**
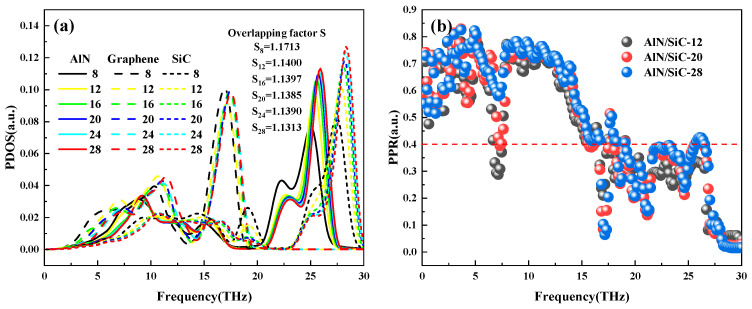
Different layers of AlN-SiC (**a**) PDOS and overlapping factor S; (**b**) *PPR*.

In order to further analyze the reason for the variation of interfacial thermal conductivity with material size, the PDOS of the aluminum nitride/graphene/silicon carbide heterogeneous interface is calculated as well as the overlapping factor S. The PDOS, overlapping factor *S*, and *PPR* images under the variation of the number of layers of AlN-SiC are shown in Figure 6, and the PDOS is analyzed for the range of the region of 0–19 THz in Figure 6a, and the analysis reveals that at 0–10 THz, SiC dominates, and the PDOS data decreases significantly with the increase of the number of layers; graphene dominates at 12–15 THz, and the PDOS picks up considerably with the rise in the number of layers, and it is found that the overlap region cannot be analyzed by the PDOS after the comparison and there is no regularity in the value of the overlapping factor S. Therefore, its *PPR* is comparatively analyzed. As shown in Figure 6b, it can be found that in the range of 0–30 THz, the *PPR* value of 28-layer AlN-SiC is the largest, then 20-layer, and finally 12-layer. This indicates that more phonons enter the localized state in the 12-layer AlN-SiC structure, resulting in a weaker interfacial phonon thermal transport compared to the 20-layer and 28-layer structures. As a result, the interfacial thermal conductance is elevated by about 57% when the number of AlN-SiC layers goes from 12 to 28.

**Figure 7:**
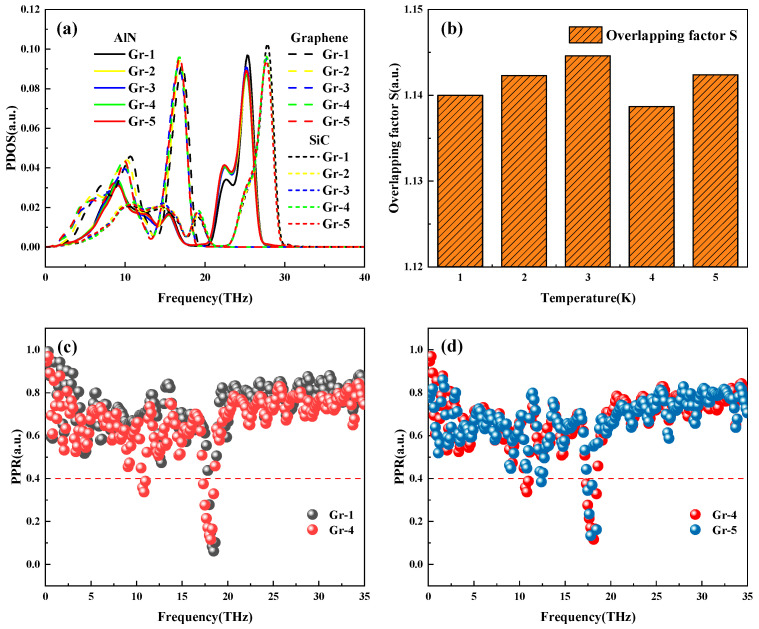
Graphene with different layers (**a**) PDOS; (**b**) overlapping factor S; (**c**) 1-layer vs. 4-layer graphene *PPR*; (**d**) 4-layer vs. 5-layer graphene *PPR*.

The values of PDOS and overlapping factor S for the variation of graphene layers are shown in Figure 7, which shows that the peak distribution of PDOS in different frequency bands does not show an obvious increasing or decreasing trend as the number of layers is increased from 1 to 5. In contrast, the overlapping factor S values show non-monotonic fluctuations in specific frequency bands. Since the PDOS and *S* values fail to clearly explain the trend of ITC, the *PPR* of graphene with different layers is further analyzed to explore the effect of phonon localization on thermal transport. It can be seen that the *PPR* value of graphene with 1 layer is generally higher than that of graphene with 4 layers in Figure 7c, which leads to a decrease in ITC of graphene with 4 layers by about 74.2% compared with that of graphene with 1 layer. When there are 4 graphene layers, the *PPR* has more phonons from the delocalized mode into the localized mode in the low-frequency range of 8–12 THz, the phonon thermal transport is blocked, and the interfacial thermal conductivity decreases. The comparison of *PPR* when graphene has 4 and 5 layers is shown in Figure 7d. Compared with 4-layer graphene, 5-layer graphene *PPR* has more phonons from localized to delocalized mode, and the *PPR* of 5-layer graphene is significantly higher than that of the 4-layer system in the critical band (0–20 THz), which is beneficial to interface heat dissipation by participating in the increase of their transporting phonon. Ultimately, the ITC of 5-layer graphene is increased by about 13.2%.

### 3.3. Impact of Defects

Due to the limitations of the preparation process, it is difficult to obtain structurally complete monolayer graphene in actual preparation, and all kinds of defects are inevitably formed in the interface, which becomes the key factor affecting the thermal conductivity of the interface of heterostructures [45]. It has been shown that the increase of defects can seriously affect the interfacial thermal conductivity of heterostructures, e.g., in diamond/graphene [36] heterostructures, ITC decreases and then gradually increases with the increase of the density of single vacancy defects in graphene; vacancy defects lead to a significant decrease of thermal conductivity in graphene/stannylene [46] heterostructures; and graphene/phosphorine [47] ITC increases with the increase of the defect rate. The effect of defects on graphene-related heterostructures is relatively complex. Therefore, this section will investigate the impact of SV (single vacancy), DV (double vacancy) and MV (mixed vacancy, which includes single and double vacancies) defects on the thermal conductivity at the interface of aluminum nitride/graphene/silicon carbide heterostructures at 300 K. The size of the heterostructure model was fixed at 12 × 1 × 12, and the interfacial thermal conductivity of the graphene layer was simulated with defect rates ranging from 0% to 25% to verify its convergence and extreme behavior.

**Figure 8:**
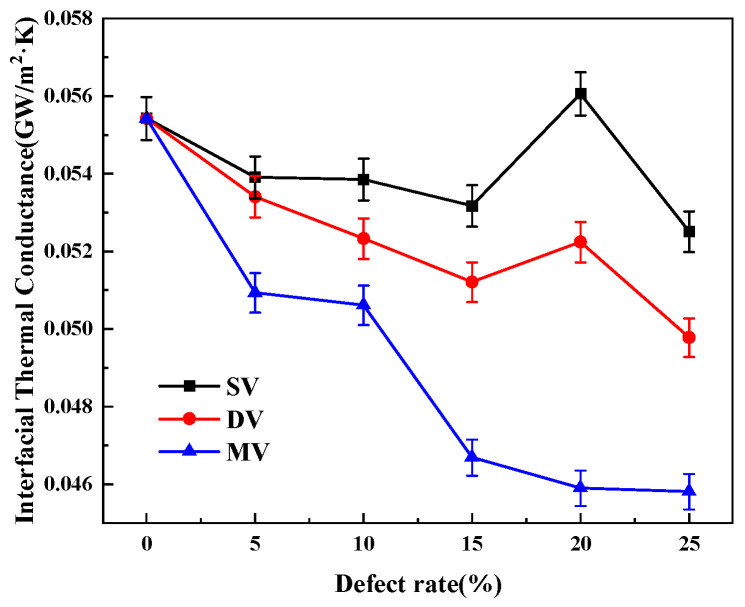
Interfacial thermal conductivity under the influence of different types of defects.

As shown in Figure 8, when the defect rate is in the range of 0–15%, the mean free range of phonons decreases due to lattice scattering induced by defects in the crystals [22]. The interfacial thermal conductance from the original 5.317 × 10^−2^ GW/m^2^K for the absence of defects decreases to 5.5096 × 10^−2^ GW/m^2^K. When the defect rate is 20%, the interfacial of SV defects thermal conductivity of SV defects increases to a peak of 5.606 × 10^−2^ GW/m^2^K and then starts to decrease to 5.251 × 10^−2^ GW/m^2^K. The trend of the interfacial thermal conductivity of DV defects is consistent with that of SV, which first decreases to 5.12 × 10^−2^ GW/m^2^K, then shows a peak of 5.224 × 10^−2^ GW/m^2^K at a 20% defect rate. Finally, the interfacial thermal conductance decreases continuously to 4.978 × 10^−2^ GW/m^2^K. The interfacial thermal conductance of MV defects decreases gradually with the increase of defect concentration to 4.6 × 10^−2^ GW/m^2^K, a decrease of 16.5%.

**Figure 9:**
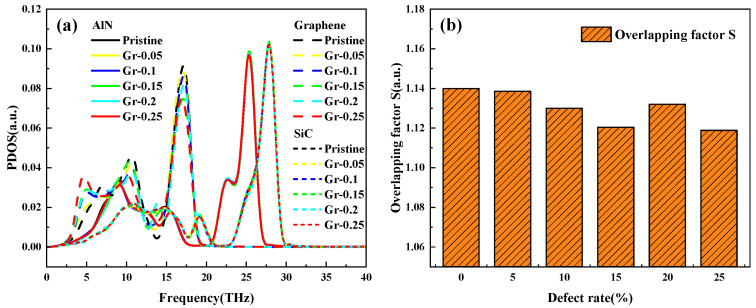
(**a**) PDOS; (**b**) overlapping factor S at each defect concentration for single vacancies.

The variation of thermal conductivity at the interface of SV and DV at a defect concentration of 20% will be analyzed next. Figure 9 shows the PDOS profiles and the variation of the overlapping factor S under a single graphene vacancy. As shown in Figure 9a, the overlap of the PDOS curves of AlN and SiC is high, indicating that AlN and SiC have no significant effect on the defect rate of the graphene layer. Therefore, only the graphene PDOS is analyzed, and three peaks of graphene PDOS are formed in the figure, of which the first peak is at 5 THz, and with the increase of graphene defect rate, the PDOS is also increased from 0.018 to 0.037, which increases the overlapping area; the second peak is at 10 THz, and there is no regular change of the PDOS with the increase of defect rate; the second peak is at 10 THz, and the PDOS is at 17 THz. At 17 THz, for the graphene PDOS formation of the last peak, the change rule is entirely consistent with the interface thermal conductivity value of the trend of PDOS with the defect rate of the first decrease, and then with the defect rate of 20% at the increase, and then the decrease. In summary, the PDOS change rule is more complex. The effect of phonon overlap on the interfacial thermal conductivity is further analyzed by calculating the overlapping factor S. Figure 9b shows that the overlapping factor S value with the defect rate of the rise of the change in line with the rule of law of the interface thermal conductivity, the analysis that when the single vacancy defect rate of 20%, phonons in the same frequency range has a higher vibration mode, phonon coupling strength enhancement so that the influence of this defect rate of the interface thermal conductivity is higher than the surrounding values.

**Figure 10:**
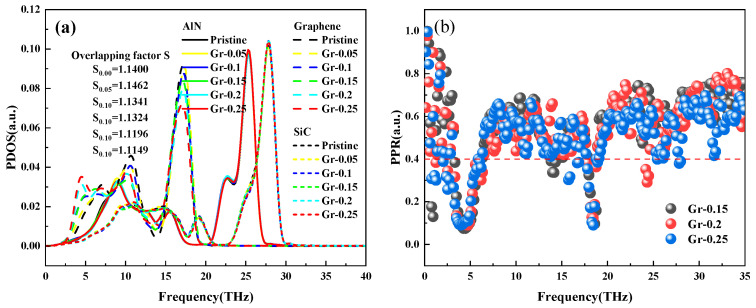
(**a**) PDOS vs. overlapping factor S at each defect concentration in double vacancies; (**b**) *PPR*.

The PDOS, overlapping factor *S*, and *PPR* of graphene at each defect concentration in the double vacancy are shown in Figure 10. Similar to the PDOS under a single vacancy in Figure 10a, the overlap rate of the PDOS curves of AlN and SiC is also high, so only the PDOS of graphene at 0–19 THz is analyzed, and the analysis reveals that there is a highly complex trend of change between the PDOS, and the overlapping factor S can not be found to have the exact change rule as that of ITC. Therefore, to prove the variation rule of ITC, the *PPR* at different defect concentrations was calculated, as shown in Figure 10b. Upon comparison, it is found that 25% versus 15% defect concentration leads to more phonon localization, phonon thermal transport is more severely hindered, and the interfacial thermal conductance exhibits a lower value.

## 4. Conclusions

In this study, we employ a nonequilibrium molecular dynamics approach to systematically investigate the thermal transport regulation mechanism of the aluminum nitride/graphene/silicon carbide heterointerface. We also unveil the influence of three key factors—temperature, size, and interfacial defects on interfacial thermal conductance through PDOS, S-factor, and *PPR* studies. The results indicate the following:

1. The significant increase in ITC with increasing temperature is attributed to the fact that more phonons are excited at high temperatures, which enhances the interfacial phonon coupling and improves the thermal transport capacity, and the interfacial thermal conductance increases with the coupling strength factor.
2. The size of the heterostructure has an essential effect on the ITC; the increase in the number of AlN/SiC layers increases the interfacial thermal conductance, which is due to the rise in the number of layers effectively reducing the interfacial scattering and improving the phonon transport efficiency across the interface. In contrast, the increase in the number of graphene layers exacerbates the localization effect of the phonons, resulting in a continuous decrease in the ITC up to 4 layers. The increase in the ITC at 5 layers is due to the transition of the phonons from localized to the delocalized mode, increasing the heat transfer phonons.
3. Structural defects in graphene usually weaken the interfacial thermal conductivity. Still, when the single vacancy defect rate reaches 20%, there may be a localized rebound in ITC, which may be related to the optimization of phonon mode matching at a specific defect concentration. The most important reason for this condition in the double vacancy defect concentration is the transition of the phonons from the localized to the delocalized state at this concentration of defects, which makes the heat-transferring phonon increase, and consequently, the ITC is enlarged.

The results of this study reveal the microscopic heat transport mechanism at the heterogeneous interface of aluminum nitride/graphene/silicon carbide, which provides a theoretical basis for optimizing the “self-heating effect” of the interface and provides scientific guidance for the development of high-performance thermal interface materials and the heat dissipation management of power electronic devices.

## Figures and Tables

**Table 1:** Lattice parameters and cell expansion factor.

Orientations	Aluminum Nitride		Graphene		Silicon Carbide	
	Original Cell Parameters (Å)	Enlargement Factor	Original Cell Parameters (Å)	Enlargement Factor	Original Cell Parameters (Å)	Enlargement Factor
X	5.419	7	4.260	9	5.330	7
Y	3.129	8	2.460	10	3.070	8
Z	2.508	6	-	-	2.512	6

**Table 2:** Lennard-Jones parameters.

Parameters	ε (eV)	σ (Å)
Al-C	0.030755	3.010
Al-Si	0.011300	3.118
Si-N	0.012705	3.375
N-C	0.003690	3.346

## Data Availability

The original code and data processing cannot be shared; all data is included in the article.
